# Risk, Characteristics and Biomarkers of Cytokine Release Syndrome in Patients with Relapsed/Refractory AML or MDS Treated with CD3xCD123 Bispecific Antibody APVO436

**DOI:** 10.3390/cancers13215287

**Published:** 2021-10-21

**Authors:** Fatih M. Uckun, Justin Watts, Alice S. Mims, Prapti Patel, Eunice Wang, Paul J. Shami, Elizabeth Cull, Cynthia Lee, Christopher R. Cogle, Tara L. Lin

**Affiliations:** 1 Department of Regulatory Affairs and Clinical Research, Aptevo Therapeutics, Seattle, WA 98121, USA; leec@apvo.com; 2 Immuno-Oncology Program, Ares Pharmaceuticals, St. Paul, MN 55110, USA; 3 Division of Hematology, Department of Medicine, Sylvester Comprehensive Cancer Center, University of Miami, Miami, FL 33136, USA; jxw401@miami.edu; 4 The James Cancer Hospital, The Ohio State University Comprehensive Cancer Center, Columbus, OH 43210, USA; Alice.Mims@osumc.edu; 5 Harold C. Simmons Comprehensive Cancer Center, Department of Internal Medicine, Division of Hematology-Oncology, University of Texas Southwestern Medical Center, Dallas, TX 75390, USA; prapti.patel@UTSouthwestern.edu; 6 Roswell Park Comprehensive Cancer Center, Department of Medicine, Buffalo, NY 14263, USA; Eunice.Wang@RosewellPark.org; 7 Division of Hematology and Hematologic Malignancies, Huntsman Cancer Institute at the University of Utah, Salt Lake City, UT 84112, USA; paul.shami@utah.edu; 8 Greenville Health System, Institute for Translational Oncology Research, Greenville, SC 29605, USA; Liz.Cull@prismahealth.org; 9 Department of Medicine, Division of Hematology & Oncology, University of Florida, Gainesville, FL 32610, USA; Christopher.Cogle@medicine.ufl.edu; 10 Division of Hematologic Malignancies and Cellular Therapeutics, The University of Kansas Cancer Center, Westwood, KS 66205, USA; tlin@kumc.edu

**Keywords:** AML, CD123, bispecific antibody, T-cells, leukemia, clinical study, APVO436

## Abstract

**Simple Summary:**

Cytokine release syndrome is a potentially life-threatening complication of therapy with T-cell engaging bispecific antibodies. Here we evaluated the risk, characteristics and biomarkers of treatment-emergent cytokine release syndrome in patients with relapsed/refractory acute myeloid leukemia or myelodysplastic syndrome who received weekly intravenous infusions of the CD3xCD123 bispecific antibody APVO436. Cytokine release syndrome was encountered in 10 of 46 patients (21.7%) treated with APVO436 with a cumulative Grade 3/4 cytokine release syndrome incidence of 8.7%. Cytokine profiling in patients who developed cytokine release syndrome after APVO436 infusion indicated that the predominant cytokine in this inflammatory cytokine response was IL-6. The findings from this research provide new insights regarding the biology and effective management of cytokine release syndrome in leukemia patients treated with T-cell redirecting bispecific antibodies.

**Abstract:**

We evaluate the risk, characteristics and biomarkers of treatment-emergent cytokine release syndrome (CRS) in patients with relapsed/refractory acute myeloid leukemia (AML) or myelodysplastic syndrome (MDS) who received APVO436 during the dose-escalation phase of a Phase 1B study (ClinicalTrials.gov, identifier: NCT03647800). Of four patients who developed Grade ≥ 3 CRS, two received steroid prophylaxis. The dose level, gender, race, obesity, or baseline hematologic parameters in peripheral blood did not predict the risk of CRS. Patients with a higher leukemia burden as determined by a higher total WBC, higher percentage of blasts in bone marrow, or higher percentage of blasts in peripheral blood (by hematopathology or immunophenotyping) did not have a higher incidence of CRS. There was an age difference between patients who did versus patients who did not develop CRS (72.9 ± 1.6 years (Median 73.5 years) vs. 63.3 ± 2.3 years (Median: 65.0 years), which was borderline significant (*p* = 0.04). Premedication with steroids did not eliminate the risk of CRS. Cytokine profiling in patients who developed CRS after APVO436 infusion indicates that the predominant cytokine in this inflammatory cytokine response was IL-6. APVO436-associated CRS was generally manageable with tocilizumab with or without dexamethasone. Notably, the development of CRS after APVO436 therapy did not appear to be associated with a response. The prolonged stabilization of disease, partial remissions and complete remissions were achieved in both patients who experienced CRS, as well as patients who did not experience CRS after APVO436 infusions.

## 1. Introduction

An urgent unmet medical need in acute myelogenous leukemia (AML), the most common form of adult acute leukemia, is to salvage relapsed or refractory R//R patients who have a dismal prognosis with <10% surviving five years [1,2,3,4,5,6,7,8,9,10]. Biotherapeutic agents, including CD3-engaging bispecific antibodies (BiAb), may provide the foundation for new and effective treatments against R/R AML [9,10]. CD3-engaging BiAb recruit cytotoxic T-cells (CTL) to the close vicinity of AML cells to create “cytolytic synapses” which triggers a CTL-mediated destruction of AML cells [11,12,13,14,15,16]. AML-directed CD3-engaging BiAb act as agonists and activate T-cells in the presence of tumor cells expressing the target tumor-associated antigen, which can lead to an excessive T-cell activation with the release of inflammatory cytokines and the development of potentially life-threatening systemic inflammation, known as cytokine release syndrome (CRS) [17,18,19,20,21].

The α-chain of the interleukin-3 (IL-3) receptor, also known as the CD123 antigen, is broadly expressed on AML cells [22,23,24,25]. APVO436 is a recombinant CD3-engaging BiAb designed to redirect CTLs in a major histocompatibility complex (MHC)-independent manner to CD123-expressing AML cells [26,27]. Dissimilar to previously described bispecific antibody fragments, APVO436 with its ADAPTIR format binds bivalently to both CD123 and CD3, yet does not cross-link and activate T-cells without a target present [26,27]. APVO436 also incorporates a modified antibody Fc region that improves serum stability, but does not cross-link T-cells or target cells through Fc gamma receptors such as CD16 or CD64 [26]. Preclinical data comparing APVO436 with a CD3xCD123 dual-affinity re-targeting (DART) molecule, MGD006, evaluating T-cell activation, proliferation, cytotoxicity and cytokine secretion, showed that APVO436 and MDG006 are both effective at stimulating a tumor-directed immune response by inducing a comparable T-cell activation, proliferation and cytotoxicity [27]. However, in these preclinical studies, APVO436 induced lower levels of several T-cell cytokines, including interferon gamma (IFNγ), interleukin-2 (IL-2), interleukin-(IL-6), tumor necrosis factor alpha (TNFα) and several additional cytokines, suggesting a potential safety advantage with APVO436 [27]. This is reminiscent of the published data on the first generation ADAPTIR candidate, APVO414, showing a reduced cytokine release upon T-cell engagement compared to another bispecific format [28].

In a Phase 1B dose-finding study in relapsed/refractory AML and MDS patients (ClinicalTrials.gov identifier: NCT03647800), this CD3-engaging bispecific antibody exhibited promising single-agent activity (29). While the safety profile was overall favorable and the maximum tolerated dose (MTD) was >60 mcg/week, some patients experienced CRS as a potentially life-threatening treatment-emergent complication [29]. Within the confines of a small patient and heterogeneous patient population, the CRS rate of 21.7% in the Phase 1B study of APVO436 appeared to compare favorably with the reported CRS rates for the anti-AML bispecific antibodies: CD33xCD3 bispecific antibody AMG330 (67%—Clinicaltrial.gov; identifier: NCT#02520427), CD33xCD3 bispecific antibody AMG673 (63%—Clinicaltrial.gov; identifier: NCT03224819), CD3xCD123 bispecific, DART antibody Flotetuzumab (96%) [11] and CD3xCD123 bispecific antibody Vibecotamab (XmAb14045) (58%) [30]. If these preliminary results pertaining to the tolerability of APVO436 and low CRS rate are confirmed in an additional clinical evaluation of APVO436, APVO436 may emerge as a clinically meaningful adjunct to existing AML drugs. Nevertheless, CRS was the second most common APVO436-related AE causing an interruption of infusions, dose delays, dose reductions as well as the discontinuations of protocol therapy [29].

In order to mitigate the risk of CRS in APVO436-receiving AML/MDS patients, a better understanding of the mechanism and kinetics of CRS after APVO436 administration, its predictive clinical and laboratory biomarkers as well as the effectiveness of available CRS-treatment algorithms in preventing and/or managing APVO436-asociated CRS will be of paramount importance. Here, we extend our recently published observations regarding the tolerability of APVO436 and APVO436-associated CRS [29] and report, for the first time, the kinetics of cytokine responses in APVO436-treated patients who developed moderate–severe CRS, an effective CRS management algorithm, the clinical significance as well as risk factors for CRS.

## 2. Materials and Methods

### 2.1. Investigational Medicinal Product

APVO436 is a humanized BiAb that binds to both CD123 and CD3 [26]. It is a homodimeric antibody comprised of two sets of binding domains linked to a human immunoglobulin (Ig) G1 fragment crystallizable (Fc) domain [29]. The CD123 binding domain is a fully human single-chain variable fragment (scFv) directed against human CD123. The CD3 binding domain is a humanized scFv derived from a murine antibody that binds human CD3. APVO436 drug substance (DS) was produced in accordance with current Good Manufacturing Practices (GMP) by the contract manufacturer KBI Biopharma, Inc (Durham, NC, USA) using a master cell bank transfected with an expression plasmid encoding APVO436.

### 2.2. Study Design and Patients

The clinical trial was registered in the clinical trial database ClinicalTrials.gov with the identifier number NCT03647800. In total, 58 patients were screened and 46 R/R AML/MDS patients who met the study eligibility criteria were enrolled. The details of the study design and patient eligibility criteria were recently reported [29].

### 2.3. Study Conduct

The open-label Phase 1 study was performed at the following 10 centers in the US as an open-label study sponsored by Aptevo Therapeutics [29]. The starting dose in Cohort 1 was 0.3 mcg (~0005 mcg/kg for a 60 kg patient), which was the Minimum Anticipated Biological Effect Level (MABEL) [31]. The assigned weekly target dose levels for Cohorts 2–10 ranged from 1 mcg to 60 mcg according to a 3 + 3 dose escalation scheme [29].

### 2.4. Ethics Statement and Study Approval

The study protocol was approved by the WCG-Central Institutional Review Board (IRB) (OHRP/FDA registration number: IRB00000533) and the local IRB at participating centers [29]. The Central IRB-approved study/protocol number was 20181730. The study was performed in compliance with the International Conference on Harmonization (ICH) guidelines for Good Clinical Practice (ICHE6/GCP). Each patient provided a written informed consent (ICF) prior to enrollment.

### 2.5. Grading and Management of Cytokine Release Syndrome (CRS)

CRS was defined and graded and managed, as detailed in Tables S1 and S2, respectively [21]. We also applied the 2019 American Society for Transplantation and Cellular Therapy (ASTCT) Consensus Grading criteria [32].

### 2.6. Measurement of Serum Cytokine Levels and Flow Cytometry

The MESO Scale Discovery (MSD) U-PLEX assay platform and an MSD MESO QuickPlex SQ 120 Reader Instrument (MESO Scale Diagnostics, Rockville, MD, USA) were used in a Central Laboratory setup for measurement of serum levels of the proinflammatory cytokines interleukin-5 (IL-5), IL-6, interleukin-10 (IL-10), interleukin-17A (IL-17A), IFN-γ, monocyte chemoattractant protein 1 (MCP-1) and TNF-α by electrochemiluminescence in duplicate serum samples from 3 patients with CRS (1 with Grade 2 CRS, 1 with Grade 3 CRS and 1 with Grade 4 CRS) [29]. In one additional case with Grade 3 CRS, serum IL-6 levels were determined by the local laboratory. The longitudinal changes in serum cytokine levels were evaluated in patients with CRS by comparing the mean concentrations for each time point to baseline concentrations. Immunophenotyping was performed on cryopreserved peripheral blood mononuclear cells from patients by standard flow cytometry using a BD LSR II flow cytometer (BD Biosciences, San Jose, CA, USA) and FACSDiva Software Version 8.0.2 fluorochrome-labeled monoclonal antibodies reactive with CD5 (anti-human CD5, clone REA782 (PE-Vio770), CD45 (anti-human CD45, Clone H130, V500, BD Biosciences #560777), CD34 (anti-human CD34, Clone REA1164, Vio Bright 515, Miltenyi Biotec #130-120-517, Auburn, CA, USA), CD38 (anti-human CD38, clone HIT-2, BV605, Biolegend #303532, San Diego, CA, USA), and CD123 (anti-human CD123, Clone 9F5, AF647, BD Biosciences #563599) antigens.

### 2.7. Statistical Analyses

Standard statistical methods were applied for the analysis of the clinical data. Survival data were analyzed by the Kaplan–Meier method using the GraphPad Prism 9 statistical program (GraphPad Software, LLC, San Diego, CA, USA). Log-rank statistics were used to compare the differences between patient subgroups. 

## 3. Results

### 3.1. Cytokine Release Syndrome and Its Predictors

APVO436 exhibited an overall favorable safety profile with an acceptable tolerability and manageable treatment-emergent adverse events (AEs) in 46 patients with relapsed/refractory AML/MDS who were treated on the Phase 1B study 5001 [29]. The MTD was not reached at a weekly dose of 60 mcg which was tolerated by all four patients enrolled without any dose-limiting toxicities (DLTs) or Grade 3–4 treatment-emergent AEs. The single dose recommended Phase 2 dose (RP2D) level has been identified as an 18 mcg flat dose (Cohort 6; ~0.2 mcg/kg based on the body weights of the patients enrolled), which was 30% of the Cohort 10 dose level [29].

Grade 3–4 CRS was the 6th most common Grade ≥3 AE, following febrile neutropenia, anemia, hyperglycemia, decreased platelet count and sepsis by the Medical Dictionary for Regulatory Activities (MedDRA) preferred term (PT) occurring in patients treated with APVO436 in Study 5001 regardless of any relationship with the study drug APVO436, and it was encountered in four patients (8.7%) [29]. CRS was reported as a serious adverse event (SAE) in 7 (70%) of the 10 patients who developed CRS (Table 1). Table S3 shows the listing of all AE leading to dose modifications of APCO436. CRS led to dose interruptions in four patients, a dose reduction in one patient and permanent discontinuation of the study drug in one patient (Tables S4 and S5). Only 2 of the 46 patients experienced DLT and it was related to CRS in both patients (Tables S4 and S5). 

Premedication with steroids (dexamethasone) did not eliminate the risk of CRS. Of four patients who developed Grade ≥3 CRS, two had received steroid prophylaxis (Table 1). Notably, CRS did not show an apparent dose relationship. The average dose levels were 0.28 ± 0.21 (median: 0.19) µg/kg for those patients who developed CRS and 0.28 ± 0.27 (Median: 0.20) µg/kg for those who did not develop CRS (*p* = 0.97). A total of 6 of 18 patients treated in cohorts 4–6 and 3 of 17 patients treated in cohorts 7–10 developed CRS (Table 1). In total, 2 of 18 patients from cohorts 4 to 6 and 1 of 17 patients from cohorts 7 to 10 developed ≥Grade 3 CRS (Table 1). There was a borderline significant age difference between patients who did versus patients who did not develop CRS (72.9 ± 1.6 years (Median 73.5 years) vs. 63.5 ± 2.3 years (Median: 65.0 years) (*p* = 0.04). APVO436 dose, gender or race did not affect the incidence of CRS. Importantly, the percentage of T-cells in peripheral blood did not predict CRS (Table 2). There was a statistically insignificant (*p* = 0.1) trend towards a higher absolute lymphocyte count for patients who experienced CRS. Patients with a higher leukemia burden as determined by a higher total WBC, higher percentage of blasts in bone marrow, or higher percentage of blasts in peripheral blood (by hematopathology or immunophenotyping) did not have a higher incidence of CRS (Table 2).

Obesity is considered a significant contributor to inflammatory cytokine production [31,32], and it was reported as a risk factor for both CRS and neurotoxicity in patients treated with IL-2 [33]. We, therefore, sought to determine if obesity was a risk factor for APVO436-related CRS. BMI values were available for 45 of the 46 patients in the safety population. While 9 of 32 non-obese patients (28.1%) developed CRS, only 1 of 13 obese patients (7.7%) developed CRS, consistent with a trend towards a lower risk of CRS for obese patients that was not statistically significant (*p* = 0.14). Only one of the 10 patients who developed CRS was obese (212-0005), and he had Grade 1 CRS with a total duration of 2 days without any use of tocilizumab (Table 1). The BMI of the 10 patients who developed CRS was not higher than the average BMI of patients who did not experience CRS (Table 2). Hence, our results did not indicate that obesity is a significant contributing factor to treatment-emergent CRS in APVO436-receiving patients.

### 3.2. Serum Cytokine Profiles of Patients Who Developed CRS

**Figure 1:**
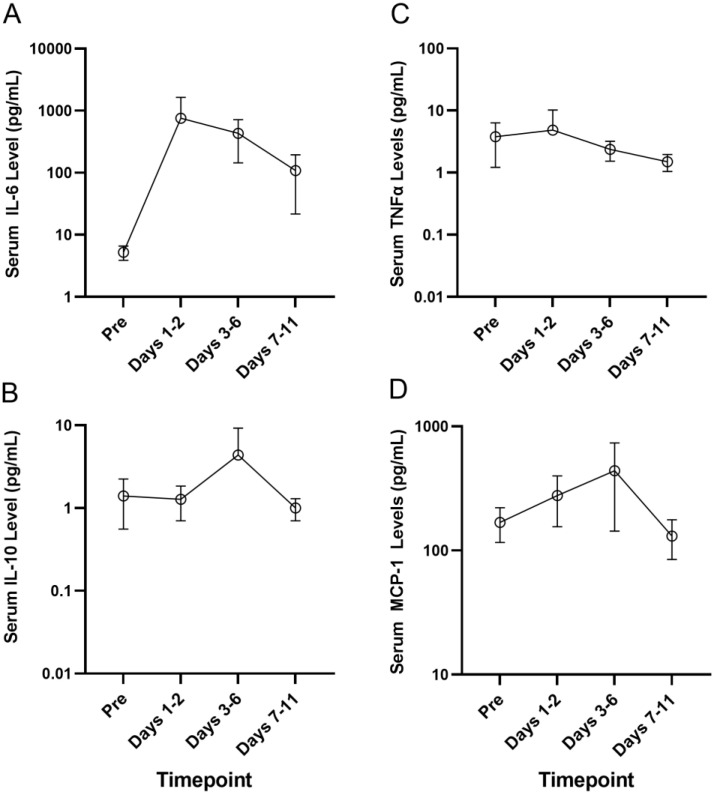
Serum cytokine levels of patients who developed CRS after APVO436. The MSD U-PLEX assay platform was used for measurement of serum levels of the proinflammatory cytokines IL-5, IL-6, IL-10 and TNF-α by electrochemiluminescence in serum samples from a select group of 4 primary AML patients who experienced Grade 2–4 CRS. Serum samples obtained pretreatment and at multiple timepoints after initiation of APVO436 treatment were used to understand the longitudinal changes in serum cytokine levels. The results are also presented in Table S6. See text for detailed discussion of the results. (**A**) Serum IL-6 levels (**B**) Serum IL-10 levels (**C**) Serum TNFα levels (**D**) Serum MCP-1 levels.

The MSD U-PLEX assay platform was used for the measurement of serum levels of the proinflammatory cytokines by electrochemiluminescence in serum samples from a select group of four primary AML patients who developed Grade 2–4 CRS, including 214-0002 in Cohort 1 who developed Grade 3 CRS, 217-0002 in Cohort 4 who developed Grade 4 CRS, 203-0004 in Cohort 6A who developed Grade 3 CRS and 214-0011 in Cohort 7 who developed Grade 2 CRS. Serum samples obtained pretreatment and at multiple timepoints after the initiation of APVO436 treatment were used to monitor the longitudinal changes in serum levels of inflammatory cytokines. There was a marked and sustained increase in serum IL-6 levels detected between days 1 and 6 post exposure to APVO436 (Table S6, Figure 1). Within 1–2 days, following the first dose of APVO436, the mean serum IL-6 concentration was elevated 145-fold over the baseline (755 vs. 5.2) and at the end of one week it was still elevated 83-fold over the baseline. By comparison, the surge in the serum levels of IL-5, IL-10, MCP-1 and TNF-α were transient with a mild–moderate increase over baseline levels (Table S6, Figure 1). Levels of IL-17A and IFN-γ did not show a significant or consistent elevation (Table S6, Figure 1). These results indicated that the APVO436-related CRS in AML patients was a largely IL-6-dominated systemic inflammatory process. Six patients received tocilizumab as part of their standard of care CRS management, three patients had dose reductions and/or dose delays, two patients had a temporary interruption of their APVO436 therapy plan and in three patients (214-0002, 203-0003, 217-0002) with Grade 2–4 CRS whose CRS was reported as an SAE, APVO436 was permanently discontinued. No changes to dose or schedule were determined in 219-0003 who developed a Grade 1 CRS that resolved within a day (Table 1).

### 3.3. Clinical Responses to APVO436 in Patients Who Developed CRS

Of the 39 R/R AML patients, 34 were evaluated for a response. Twelve patients (35.3%) had progressive disease (PD) and died of leukemia between 29 and 70 days (median: 43 days). A total of 22 of these 34 patients (64.7%) had stable disease (SD) as their best overall response [30]. In 8 of these 22 patients, SD was achieved between 31 and 75 days after study entry and lasted ~3 months or longer [29].

**Figure 2:**
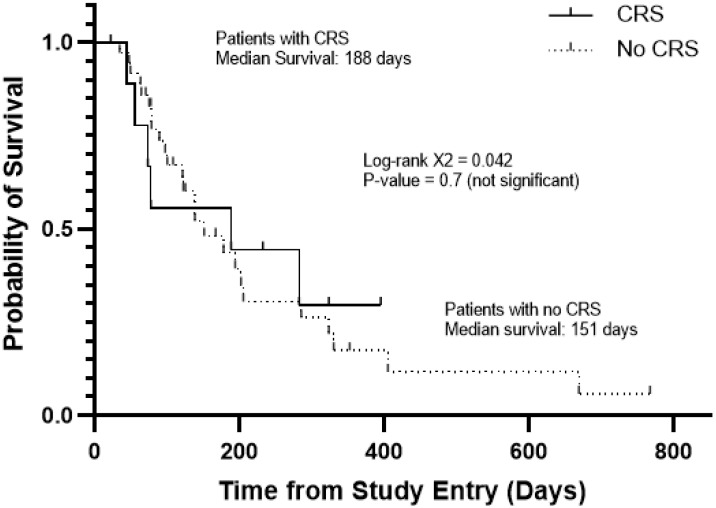
Survival outcome of AML/MDS patients according to development of CRS in the course of their APVO436 therapy. Depicted are the overall survival curves of the 10 patients who developed CRS and 36 patients who did not. See also Table 2.

Among the patients with favorable responses, four experienced CRS and four did not. APVO436-related CRS was not required for clinically meaningful responses in R/R AML patients (Table 3), and it did not affect the survival outcome (Figure 2). The median survival was 188 days for patients with CRS and 151 days for those without CRS (Log-rang X^2^ = 0.042, *p* = 0.7) (Figure 2). Prolonged stabilization of disease, partial remissions and complete remissions were achieved in both patients who experienced CRS as well as patients who did not experience CRS after APVO436 infusions.

## 4. Discussion

A common complication of bispecific antibody treatments is CRS [17,18,19,20,21]. For example, the human bispecific antibody AMG330 binds CD33 antigen on AML cells and CD3Ԑ on T-cells. In an open-label Phase 1 study (Clinicaltrial.gov, identifier: NCT02520427), AMG330 was given at doses ranging from 0.5 to 720 μg/d in the manner of continuous IV infusion among 55 patients with R/R AML. AMG 330-related AEs included CRS (67%; Grade ≥ 3 in 13%) as the most frequent AEs. Similarly, CRS was observed in 63% of AML patients treated with AMG673, a new version of AMG330 (Grade ≥ 3 in 18%; Clinicaltrial.gov, identifier: NCT03224819) [34]. Flotetuzumab (MGD006) is a bispecific DART antibody reactive with both a CD3 antigen on T-cells and a CD123 antigen on AML cells. This CD3-engaging BiAb exhibited promising single-agent activity in therapy-refractory AML patients with primary induction failure as well as patients with an early first relapse. CRS was observed in all AML patients treated with Flotetuzumab [11] and 58% of AML patients treated with Vibecotamab (XmAb14045), another CD3xCD123 BiAb [35]. By comparison, only 10 of 46 patients (21.7%) treated with APVO436 developed CRS. 

IL-6 is one of the driving pro-inflammatory cytokines that contributes to CRS and its pulmonary, cardiovascular, renal and neurologic complications [17,18,19,20,21,36,37]. Cytokine profiling in patients who developed CRS after APVO436 infusion indicated that the predominant cytokine in this inflammatory cytokine response was IL-6, which was in agreement with our current knowledge regarding CRS that occurs in the context of BiAb therapy [14,15,16,38,39,40]. Within 1–2 days following the first dose of APVO436, the mean serum IL-6 concentration was elevated 145-fold over the baseline (755 vs. 5.2) and at the end of one week it was still elevated 83-fold over the baseline. In most cases, CRS events were transient and medically manageable with standard of care, including the use of dexamethasone and anti-IL-6:IL-rR antibody tocilizumab or anti-IL-6 antibody siltuximab (antibody against IL-6). One patient who developed Grade 2 CRS died due to complications from acute renal failure. Notably, patients who developed CRS after APVO436 therapy were not more or less likely to have a favorable response. As patients received dexamethasone as a premedication and for the treatment of CRS, these results suggest that dexamethasone does not prevent favorable responses to APVO436 at the applied dose level and schedule.

## 5. Conclusions

APVO436-related CRS was encountered in 10 of 46 patients (21.7%) treated with APVO436 with a cumulative Grade 3/4 CRS incidence of 8.7%. Cytokine profiling in patients who developed CRS after APVO436 infusion indicated that the predominant cytokine in this inflammatory cytokine response was IL-6. APVO436-associated CRS was generally manageable with standard of care and resolved rapidly with the administration of tocilizumab at standard doses combined with dexamethasone. APVO436-related CRS was not required for clinically meaningful responses in R/R AML patients, and it did not affect their survival outcome. The prolonged stabilization of disease, partial remissions and complete remissions were achieved in both patients who experienced CRS as well as patients who did not experience CRS after APVO436 infusions.

## Figures and Tables

**Table 1:** Summary tabulation of CRS data (all grades) from study 5001 dose-escalation phase using 2019 ASTCT criteria.

Patient No. ***	Steroid Premeds	DL	Start Date	Grade	SAE (Yes/No)	Neuro-Toxicity	Total Duration (Days)	Relatedness with APVO436	AE Outcome	Changes to Drug Dose or Schedule	Tocilizumab Yes/No
214-0002	No	1	C6D1	3	Yes	No	8	Related	Resolved	DPD	Yes
203-0003 **	Yes	4	C2D1	2	Yes	Yes	>12	Related	Fatal (2° ARF, Grade 5)	DPD	Yes
213-0005	Yes	4	C1D3	2	Yes	No	4	Related	Resolved	DD	No
217-0002	Yes	4	C1D2	4	Yes	No	9	Related	Partially Resolved	DPD	Yes
215-0004	Yes	6A	C6D17	1	No	Yes	2	Related	Resolved	DR/DD	No
212-0005	No	6A	C3D5	1	No	No	2	Related	Resolved	DR/DD	No
203-0004	No	6A	C1D3	3	Yes	No	10	Related	Resolved	DR/DD	Yes
214-0011	Yes	7	C5D1	2	Yes	Yes	2	Related	Resolved	TI	Yes
219-0003	No	8	C2D1	1	No	No	1	Related	Resolved	None	No
219-0005 *	Yes	A	C1D1	3	Yes	No	2	Related	Resolved	TI; pt switched to DL8	Yes

TI: temporarily interrupted (including interruption of infusion or discontinuation of infusion for the day of the AE); DPD: drug permanently discontinued; DD: next dose delayed; CRS: cytokine release syndrome; ARF: acute renal failure; 2°: secondary to; * the Grade 3 CRS observed in Patient 219-0005 from DL-A occurred after the patient received the first dose on an exploratory dose-intense regimen DL-A (Cohort A; intended dosing: daily × 4: 6–6-12–18 mcg during week 1). Specifically, patient received 6 mcg of APVO436 on day 1 and developed Grade 3 CRS during the second infusion. The cumulative dose infused was 8.5 mcg. CRS was complicated by non-ST elevation myocardial infarction (N-STEMI). Patient’s CRS and cardiac function fully recovered and patient was switched to the weekly regimen and entered into Cohort 8. Patient experienced no further CRS episodes while receiving therapy on DL8. ** Patient 203-0003 in DL4 (9 mcg/week) had a Grade 2 CRS (hypotension not requiring vasopressors) (hypotension developing on 11 December 2019, which was C2D1). This patient had a previous history of hypoxic respiratory failure and bilateral pneumonia with bilateral pleural effusions, an anterior mediastinal hematoma secondary to thrombocytopenia and pulmonary hypertension predating the enrollment in the 5001 study. This patient developed treatment-emergent infection/duodenitis, cellulitis at the site of a bone marrow biopsy, subacute splenic infarct, DIC, progressive leukemia and fatal (Grade 5) acute kidney failure (date of death: 25 December 2019). Renal consult was obtained and multiple causes were ascribed to the prerenal kidney failure experienced by patient: Lasix, decreased albumin, high dose steroids. The CRS and subsequent death from acute kidney injury were considered by the investigator to be related to APVO436. The cause of the kidney injury, while thought to be multifactorial, and temporally distinct from the last dose, was considered possibly related by the investigator because of the potential contribution from CRS. *** One patient in DL6A, 219-0002, developed fever and hypotension 6 days after C2D22; while these signs could technically be consistent with Grade 2 CRS, site PI reported them as “not related” to APVO436. Patient had a bone marrow biopsy on the same day and was found to have progressive leukemia with a fully packed bone marrow (98% cellularity, 80% leukemic blasts). CRS was not always associated with concomitant or delayed neurotoxicity. Seven of ten patients with CRS did not develop neurotoxicity (Table 1). Only 3 patients developed both CRS and neurotoxicity. One patient (203-0003 in Cohort 4, Table 1) with Grade 2 CRS with evidence of neurotoxicity, subsequently, developed, despite the use of tocilizumab, acute kidney failure with fatal outcome that was likely triggered by APVO436-related CRS. Notwithstanding the fact that it is a potentially life-threatening complication and was associated with a fatal outcome in 1 of the 46 patients in this study, CRS did not significantly affect the overall survival outcome of the safety population. The average survival times were 169.1 ± 42.1 days for patients who developed CRS and 173.9 ± 27.2 days for the remainder of patients (*p* = 0.9, Table 2).

**Table 2:** Predictors of CRS and its impact on survival.

Parameter	CRS	No CRS	*p*-Value
Age	Mean: 72.9 ± 1.6 95% CI: 69.31, 76.49 Median: 73.50 Range: 66.00–81.00	Mean: 63.3 ± 2.3 95% CI: 58.8, 68.1 Median: 65.00 Range: 18.00–82.00	0.04
Gender	Male: 70% Female: 30%	Male: 47.2% Female: 52.8%	0.2
Race	C: 70% HL:10% W-HL: 10% A: 10% B: 0%	C: 69.4% HL: 2.8% W-HL: 13.9% A: 2.8% B: 8.3%	0.6
Dose (µg/kg^+^)	Mean: 0.28 ± 0.07 95% CI: 0.11, 0.44 Median: 0.19 Range: 0.0040–0.615	Mean: 0.28 ± 0.04 95% CI: 0.19, 0.37 Median: 0.20 Range: 0.003–0.990	1.0
BMI (kg/m^2^)	Mean: 25.5 ± 1.426 95% CI: 22.3, 28.7 Median: 26.1 Range: 18.3–31.7 (BMI ≥ 30 = 10%; BMI < 30 = 90%)	Mean: 27.5 ± 1.1 95% CI: 25.2, 29.8 Median: 26.5 Range: 16.8–44.4 (BMI ≥ 30 = 34.3%; BMI < 30 = 65.7%)	0.4 (0.2)
Survival Time (ICF–Time of Death or Hospice Transfer)	Mean: 169.1 ± 42.1 95% CI: 73.8, 264.4 Median: 132.5 Range: 22–395	Mean: 173.9 ± 27.2 95% CI: 118.6, 229.1 Median: 121.5 Range: 34–767	0.9
ALC ×10^3^/µL	Mean: 1.3 ± 0.4 95% CI: 0.4, 2.2 Median: 0.9 Range: 0.19–4.36	Mean: 0.8 ± 0.1 95% CI: 0.6, 1.1 Median: 0.6 Range: 0.01–2.56	0.1
% L	Mean: 36.9 ± 6.1 95% CI: 23.2, 50.7 Median: 33.2 Range: 10–72	Mean: 29.2 ± 3.8 95% CI: 21.5, 36.9 Median: 22 Range: 2–78	0.3
WBC ×10^3^/µL	N of Patients = 10 Mean: 4.3 ± 1.495% CI: 1.10, 7.42 Median: 1.8 Range: 0.6–13.5	N of Patients = 35 Mean: 5.2 ± 1.3 95% CI: 2.5, 7.9 Median: 2.3 Range: 0.3–42.7	0.7
% Blasts in Bone Marrow	N of Patients = 10 Mean: 35.1 ± 7.0 95% CI: 19.2, 51.0 Median: 33.5 Range: 5.0–78.0	N of Patients = 36 Mean: 34.1 ± 4.7 95% CI: 24.6, 43.6 Median: 29.0 Range: 0.0–88.0	0.9
% Blasts in Blood	N of Patients = 5 Mean: 10.0 ± 9.5 95% CI: −16.4, 36.4 Median: 0.0 Range: 0.0–48.0	N of Patients = 19 Mean: 15.9 ± 6.3 95% CI: 2.6, 29.1 Median: 0.0 Range: 0.0–93.0	0.1
CD123^+^CD34^+^ cellsin blood (% of CD45^+^)	N of Patients = 7 Mean: 8.24 ± 5.09 95% CI: −4.20, 20.69 Median: 1.48 Range: 0.016–15.39	N of Patients = 23 Mean: 12.60 ± 4.63 95% CI: 2.99, 22.21 Median: 3.59 Range: 0.005–87.14	0.6
T-Cells (% of CD45^+^)	N of Patients = 7 Mean: 37.93 ± 9.12 95% CI: 15.61, 60.24 Median: 40.81 Range: 6.25–72.40	N of Patients = 23 Mean: 27.48 ± 5.10 95% CI: 16.90, 38.06 Median: 19.92 Range: 0.32–82.50	0.3

Patients who developed CRS were compared to patients who did not develop CRS relative to several demographic and other clinical/laboratory parameters, including age, gender, race, absolute lymphocyte count (ALC), % lymphocytes, white blood cell (WBC) count, leukemia burden, as measured by % of blasts in bone marrow or blood, percentage of circulating CD34^+^CD123^+^ cells within the CD45^+^CD38^−^ blast population, % of T-cells (CD5^+^ cells) as a percentage of CD45^+^ lymphoid cells. We also compared the overall survival times of these two patient populations. Statistical analyses and descriptive statistics were performed using GraphPad Prism version 9.2.0 for Windows, GraphPad Software, San Diego, CA, USA, www.graphpad.com (accessed on 30 July 2021). Unpaired two-sample t-test was used to compare the means of the numerical variables between the two groups (patients with CRS vs. patients without CRS). Chi-square statistic was used to compare the non-numerical variables. Abbreviations: M: male; F: female; C: Caucasian; A: Asian; HL: Hispanic or Latino; B: black/African American; W-HL: white Hispanic or Latino; BMI: body mass index; µg: microgram.

**Table 3:** CRS history of APVO436-treated R/R AML patients with favorable responses.

Patient ID	Cohort	CRS	Best Overall Response
214-0002	1	+(Grade 3)	SD
214-0008	5	-	SD
215-0004	6A	+(Grade 1)	SD
212-0005	6A	+(Grade 1)	PR CR
213-0009	6B	-	PR CR
214-0011	7	+(Grade 2)	SD + PBBC-C+ >50%BMB reduction
213-0012	10	-	SD
218-0004	10	-	SD/RES

PBBC-C: Clearance of peripheral blood blast count; BMB: bone marrow blast count; SD: stable disease; RES: resistant disease; PR: partial remission; CR: complete remission.

## Data Availability

Will individual participant data be available (including data dictionaries)? Yes. What data in particular will be shared? Individual participant data that underlie the results reported in this article, after de-identification (text, tables, figures, and appendices). What other documents will be available? Study protocol. When will data be available (start and end dates)? Beginning 3 months and ending 5 years following article publication. With whom? Researchers who provide a methodologically sound proposal. For what types of analyses? To achieve aims in the approved proposal.

## Supplementary Materials

The following are available online at https://www.mdpi.com/article/10.3390/cancers13215287/s1, Table S1: severity grades of CRS, Table S2: protocol 5001 management guidelines for cytokine release syndrome (CRS), Table S3: listing of all adverse events leading to dose modifications of the study drug APVO436 in study 5001, Table S4: summary tabulation by MedDRA PT of all adverse events leading to dose interruption, dose reduction or drug withdrawal occurring in patients treated with APVO436 in study 5001, Table S5: listing of dose-limiting toxicities (DLTs) in study 5001, Table S6: CRS-associated changes in serum cytokine levels of APVO436-treated R/R AML/MDS patients.
