# Supplementary material for: Risk, Characteristics and Biomarkers of Cytokine Release Syndrome in Patients with Relapsed/Refractory AML or MDS Treated with CD3xCD123 Bispecific Antibody APVO436
Source: Cancers (Basel). 2021 Oct 21;13(21):5287. doi: 10.3390/cancers13215287 (PMC8582601; doi:10.3390/cancers13215287)
Supplement: Supplementary file 1 [file cancers-13-05287-s001.zip › cancers-1403529-supplementary.pdf]

Supplementary Materials: Risk, Characteristics and Biomarkers of Cytokine Release Syndrome in Patients with Relapsed/Refractory AML or MDS Treated with CD3xCD123 Bispecific Antibody APVO436

Fatih M. Uckun, Justin Watts, Alice S. Mims, Prapti Patel, Eunice Wang, Paul J. Shami, Elizabeth Cull, Cynthia Lee, Christopher R. Cogle and Tara L. Lin

Table S1. Severity Grades of CRS.

**Grade 1**
Mild reaction; infusion interruption not indicated; the infusion rate may be reduced and then increased after the event resolves.
Symptoms are not life threatening and require symptomatic treatment only, e.g., fever, nausea, fatigue, headache, myalgias, malaise
**Grade 2**
Therapy or infusion interruption indicated but responds promptly to symptomatic treatment (e.g., antihistamines, acetaminophen, narcotics, IV fluids); prophylactic medications indicated for ≤24 hours.
Symptoms require and respond to moderate intervention
Oxygen requirement < 40%, or
Hypotension responsive to fluids or low dose of one vasopressor, or
Grade 2 organ toxicity
**Grade 3**[2]
Prolonged (e.g., not rapidly responsive to symptomatic medication and/or brief interruption of infusion); recurrence of symptoms following initial improvement; hospitalization indicated for clinical sequelae (e.g., renal impairment, pulmonary infiltrates). For the purpose of this protocol, prolonged is defined as ≥6 hours.
Symptoms require and respond to aggressive intervention
Oxygen requirement ≥ 40%, or
Hypotension requiring high-dose[1] or multiple vasopressor, or
Grade 3 organ toxicity or Grade 4 transaminitis
**Grade 4**[2]
Life-threatening symptoms / consequences; pressor or ventilator support indicated
Grade 4 organ toxicity (excluding transaminitis)
**Grade 5 – death**

[1] High-dose vasopressors are defined as any of the following: noradrenaline ≥ 20 µg/min; dopamine ≥ 10 µg/kg/min; phenylephrine ≥ 200 µg/min; adrenaline ≥ 10 µg/min; if on vasopressin, vasopressin plus noradrenaline equivalent of ≥ 10 µg/min; and if on combination vasopressors (not including vasopressin), noradrenaline equivalent of ≥ 20 µg/min. [2] In the event of a severe case of CRS (Grade ≥ 3), dosing of new patients in the trial was held until the Investigators and the Safety Review Committee review and discuss the case.

Table S2. Protocol 5001 Management Guidelines for Cytokine Release Syndrome (CRS).

**Treatment of Grade 1 CRS**
If a Grade 1 CRS occurs, then the infusion rate is to be decreased by 50%. Once the AE has resolved, the infusion of study drug may be increased gradually to its original rate over 1 to 3 hours.
Evaluate for potential infectious etiology. If neutropenia and fever persist, consider broad-spectrum antibiotics.
Symptomatic management of constitutional signs and symptoms - the following are treatment options to be considered:<br>Fever: acetaminophen 650 mg (or 15 mg/kg) orally (PO) every 4 to 6 hours as needed (PRN)<br>Rigors: meperidine 25 to 50 mg IV (or 0.5 to 1 mg/kg) every 2 to 4 hours PRN<br>Pruritus: diphenhydramine 25 to 50 mg (or 1 mg/kg) PO or IV every 2 to 4 hours PRN and hydrocortisone 100 mg IV every 2 hours PRN<br>Nausea: lorazepam 0.5 to 2 mg (0.05 mg/kg) IV every 4 hours PRN, diphenhydramine 25 to 50 mg (1 mg/kg) IV every 4 hours PRN, ondansetron 8 mg IV and every 8 hours for the first 24 hours from the start of infusion, then every 8 hours PRN<br>Bronchospasm: diphenhydramine and hydrocortisone may be repeated as above; albuterol nebulizer 2.5 to 5 mg up to every 1 to 2 hours PRN

Anaphylaxis: Cessation of study drug infusion and treat per institution standards
* In all future studies of APVO436, patients will receive of tocilizumab (or sarilumab) (antibodies against IL-6:IL-6R) or Siltuximab (antibody against IL-6) plus dexamethasone for any evidence of Grade ≥ 1 CRS lasting more than 30 min.
**Treatment of Grade 2 CRS**
Same as Grade 1
All patients with a Grade ≥ 2 CRS are to be hospitalized (or placed in a unit that can provide close monitoring and treatment) for management, supportive care, and observation until the reaction has resolved. An IRR is expected to resolve within 6 hours of terminating APVO436 infusion and starting symptomatic treatment.
If a Grade 2 toxicity is noted during the infusion (e.g., fevers, rigors, flushing), the infusion is to be halted, the patient assessed, and the patient treated with appropriate medications to reduce the severity of the signs and symptoms. Rigors are frequently observed with other bispecific antibodies and with monospecific antibodies. If rigors occur during APVO436 infusion, then the patient may be treated with meperidine (25 to 50 mg IV). Other symptomatic treatment may be initiated at the Investigator's discretion, including: promethazine (12.5 mg), hydrocortisone, anti-histamines, and/or acetaminophen. If the symptoms and signs resolve promptly, APVO436 administration may be re-initiated at one-half the previous IV flow rate, and if no adverse reactions after at least 2 hours the rate may gradually be increased.
Hypotension: IV fluid bolus of 500 to 1,000 mL of normal saline; repeat as necessary to maintain adequate volume. If hypotension persists after adequate volume replacement, then consider low-dose vasopressors.
Hypoxia: Supplemental oxygen
Organ toxicity: management of organ toxicities as per standard guidelines
Symptomatic management of constitutional signs and symptoms
If there are persistent Grade 2 symptoms, hypotension, hypoxia, or organ toxicity, 30 min or more hours after the discontinuation of APVO436 and the institution of symptomatic management, then consider this a case of CRS and start tocilizumab 8 mg/kg IV. At this point also consider the use of dexamethasone 10 mg IV. If symptoms persist for more than 12 hours, then consider tocilizumab 8 mg/kg IV; the maximum dose of tocilizumab is 800 mg. Siltuximab 11 mg/kg may be considered as an alternative to tocilizumab.
* In all future dosing patient will receive of tocilizumab (or sarilumab) (antibodies against IL-6:IL-6R) or Siltuximab (antibody against IL-6) plus dexamethasone for any evidence of Grade ≥ 1 CRS lasting more than 30 min.
**Treatment of Grade 3 CRS**
Same as Grade 2
A Grade 3 reaction requires the infusion of APVO436 to be stopped immediately and aggressive supportive and symptomatic measures to be initiated; dosing is not to be resumed on the same day. Infusion-related reactions may be severe but are generally short lived and resolve within 1 to 6 hours of drug discontinuation. If the AE is worsening or is persisting more than 6 hours, then consider dexamethasone 10 to 20 mg IV plus tocilizumab 8 mg/kg IV; the maximum dose of tocilizumab is 800 mg. According to the package insert for tocilizumab (Actemera®): If no clinical improvement in the signs and symptoms of CRS occurs after the first dose, up to 3 additional doses of tocilizumab may be administered. The interval between doses should be at least 8 hours. Siltuximab 11 mg/kg may be considered as an alternative to tocilizumab.
Consider adding anti-TNF receptor treatments (infliximab, 5 mg/kg IV)
Hypotension: IV boluses and vasopressors as needed. Consider transfer to intensive care unit (ICU) and obtain an echocardiogram and monitor hemodynamics as clinically indicated.
Hypoxia: Supplemental oxygen as needed; consider non-invasive positive pressure ventilation
Organ toxicity: management of organ toxicities as per standard guidelines<br>Report the SAE; inform Investigator and medical monitor.
**Treatment of Grade 4 CRS***
Same as Grade 3
A Grade 4 reaction requires immediate and permanent discontinuation of study drug for that patient
Transfer to ICU
IV fluids, vasopressors, and hemodynamic monitoring
Supplemental oxygen
Manage any organ toxicities
Tocilizumab 8 mg/kg IV; the maximum dose of tocilizumab is 800 mg. If no clinical improvement in the signs and symptoms of CRS occurs after the first dose, up to 3 additional doses of tocilizumab may be administered. The interval between doses should be at least 8 hours. Siltuximab 11 mg/kg may be considered as an alternative to tocilizumab.
Dexamethasone 20 mg IV; Consider adding anti-TNF receptor treatments (infliximab, 5 mg/kg IV)
Patients must receive supportive care, close monitoring, and retreatment with tocilizumab and dexamethasone as appropriate.

* In all future dosing patient will receive of tocilizumab (or sarilumab) (antibodies against IL-6:IL-6R) or Siltuximab (antibody against IL-6) plus dexamethasone for any evidence of Grade ≥ 1 CRS lasting more than 30 min.

Table S3. Listing of all adverse events leading to dose modifications of the study drug APVO436 in Study 5001.

Patient No.	Cohort#	Preferred term	System Organ Class	CTCAE Grade	Relatedness with APVO436	Action Taken with APVO436	Is the Adverse Event serious?
**Adverse events leading to dose interruption of APVO436**							
214-0001	DL-1 Dose: 0.3 mcg/week	Rash	Skin and subcutaneous tissue disorders	Grade 3	Not Related	Dose Interrupted	No
215-0002		Infusion related reaction	Injury, poisoning and procedural complications	Grade 1	Related	Infusion Discontinued for Visit	No
217-0002	DL-4 Dose: 9 mcg/week	Cytokine release syndrome	Immune system disorders	Grade 3	Related	Drug Interrupted	No
		Hypoxia	Respiratory, thoracic and mediastinal disorders	Grade 3	Related	Drug Interrupted	No
215-0004	DL-6A Dose: 18 mcg/week[a]	Back pain	Musculoskeletal and connective tissue disorders	Grade 2	Related	Infusion Discontinued for Visit	No
		Chills	General disorders and administration site conditions	Grade 2	Related	Infusion Discontinued for Visit	No
		Infusion Related Reaction	Injury, poisoning and procedural complications	Grade 2	Related	Infusion Discontinued for Visit	No
		Sepsis	Infections and infestations	Grade 3	Related	Drug Interrupted	Yes
212-0005		Cytokine release syndrome	Immune system disorders	Grade 1	Related	Dose Interrupted	No
214-0011	DL-7 Dose: 24 mcg/week[b]	Chills	General disorders and administration site conditions	Grade 2	Related	Infusion Discontinued for Visit	Yes
		Cytokine release syndrome	Immune system disorders	Grade 2	Related	Temporarily interrupted	Yes
218-0001	DL-7 Dose: 24 mcg/week[b]	Infusion related reaction	Injury, poisoning and procedural complications	Grade 2	Related	Temporarily Interrupted	Yes
219-0005	DL-8 Dose: 36 mcg/week (N=6)[c]	Acute myocardial infarction	Cardiac disorders	Grade 3	Related	Infusion Discontinued for Visit	Yes
		Cytokine release syndrome	Immune system disorders	Grade 3	Related	Drug Interrupted	Yes
200-0005	DL-9 Dose: 48 mcg/week[d]	Infusion related reaction	Injury, poisoning and procedural complications	Grade 2	Related	Infusion Discontinued for Visit	No

Patient No.	Cohort#	Preferred term	System Organ Class	CTCAE Grade	Relatedness with APVO436	Action Taken with APVO436	Is the Adverse Event serious?
219-0004	DL-10 Dose: 60 mcg/week[e]	Infusion related reaction	Injury, poisoning and procedural complications	Grade 2	Related	Infusion Discontinued for Visit	No
212-0008		Confusional state	Psychiatric disorders	Grade 3	Not Related	Drug Interrupted	Yes
213-1012		Chills	General disorders and administration site conditions	Grade 1	Related	Drug Interrupted	No
		Hypoxia	Respiratory, thoracic and mediastinal disorders	Grade 1	Related	Drug Interrupted	No
213-0011	DL-A Dose: 6 mcg/week	Neurotoxicity	Nervous system disorders	Grade 1	Related	Drug Interrupted	Yes
**Adverse events leading to dose reduction of APVO436**							
212-0005	DL-6A Dose: 18 mcg/week[a]	Cytokine release syndrome	Immune system disorders	Grade 1	Related	Dose Reduced	No
215-0004		Chills	General disorders and administration site conditions	Grade 1	Related	Infusion Rate Slowed	No
**Adverse events leading to permanent discontinuation of APVO436**							
203-0001	DL-3 Dose: 3 mcg/week	Sepsis	Infections and infestations	Grade 5	Not Related	Drug Permanently Discontinued	Yes
203-0003	DL-4 Dose: 9 mcg/week	Confusional state	Psychiatric disorders	Grade 1	Related	Drug permanently discontinued	No
212-0003		Tumor lysis syndrome	Metabolism and nutrition disorders	Grade 3	Not Related	Drug Permanently Discontinued	Yes
213-0005		Pyrexia	General disorders and administration site conditions	Grade 3	Not Related	Drug Permanently Discontinued	Yes
217-0002		Cytokine release syndrome	Immune system disorders	Grade 4	Related	Drug Permanently Discontinued	Yes
		Shock	Vascular disorders	Grade 4	Related	Drug Permanently Discontinued	No
203-0004	DL-6A Dose: 18 mcg/week[a]	Pneumonia	Infections and infestations	Grade 5	Not Related	Drug Permanently Discontinued	Yes
219-0001		Supraventricular tachycardia	Cardiac disorders	Grade 3	Not Related	Drug Permanently Discontinued	No
		Delirium	Psychiatric disorders	Grade 2	Not Related	Drug Permanently Discontinued	No
219-0002		Asthenia	General disorders and administration site conditions	Grade 3	Related	Drug Permanently Discontinued	Yes

Patient No.	Cohort#	Preferred term	System Organ Class	CTCAE Grade	Relatedness with APVO436	Action Taken with APVO436	Is the Adverse Event serious?
213-0011	DL-A Dose: 6 mcg/week	Neurotoxicity	Nervous system disorders	Grade 1	Related	Drug Permanently Discontinued	Yes
		Confusional state	Psychiatric disorders	Grade 1	Related	Drug Permanently Discontinued	Yes
		Amnesia	Nervous system disorders	Grade 1	Related	Drug Permanently Discontinued	Yes

CTCAE: Common terminology criteria for adverse events; DL: Dose level

[a] Cohort 6A: 18 mcg/week [weekly step dosing in Cycle 1: 6,9,12,18 mcg]

[b] Cohort 7: 24 mcg/week [weekly step dosing in Cycle 1: 6, 12, 18, 24 mcg]

[c] Cohort 8: 36 mcg/week [weekly step dosing in Cycle 1: 6,12,18, 36 mcg]

[d] Cohort 9: 48 mcg/week [weekly step dosing in Cycle 1: 6,12,18,48 mcg]

[e] Cohort 10: 60 mcg/week [weekly step dosing in Cycle 1: 6,12,18,60 mcg]

Table S4. Summary tabulation by MedDRA PT of all adverse events leading to dose interruption, dose reduction, or drug withdrawal occurring in patients treated with APVO436 in Study 5001.

MedDRA PT	Cohorts												Total N = 46 n (%)
	**DL-1 Dose: 0.3 mcg/week (*N* = 4)**	**DL-2 Dose: 1 mcg/week (*N* = 3)**	**DL-3 Dose: 3 mcg/week (*N* = 3)**	**DL-4 Dose: 9 mcg/week (*N* = 6)**	**DL-5 Dose: 12 mcg/week (*N* = 3)[a]**	**DL-6A Dose: 18 mcg/week (*N* = 6)[b]**	**DL-6B Dose: 12 mcg/week (*N* = 3)[c]**	**DL-7 Dose: 24 mcg/week (*N* = 4)[d]**	**DL-8[2] Dose: 36 mcg/week (*N* = 6)[e]**	**DL-9 Dose: 48 mcg/week (*N* = 3)[f]**	**DL-10 Dose: 60 mcg/week (*N* = 4)[g]**	**DL-A[2] Dose: 36 mcg/week (*N* = 1)[h]**	
**Adverse events leading to dose interruption of APVO436**													
Infusion related reaction	1 (25.0%)	0	0	0	0	1 (16.7%)	0	1 (25.0%)	0	2 (66.7%)	0	0	5 (10.9%)
Chills	0	0	0	0	0	1 (16.7%)	0	1 (25.0%)	0	0	1 (25.0%)	0	3 (6.5%)
Cytokine release syndrome	0	0	0	1 (16.7%)	0	1 (16.7%)	0	1 (25.0%)	1 (16.7%)	**0**	**0**	**0**	4 (8.7%)
Hypoxia	0	0	0	1 (16.7%)	0	0	0	0	0	0	1 (25.0%)	0	2 (4.3%)
Acute myocardial infarction	0	0	0	0	0	0	0	0	1 (16.7%)	0	0	0	1 (2.2%)
Back pain	0	0	0	0	0	1 (16.7%)	0	0	0	0	0	0	1 (2.2%)
Neurotoxicity	0	0	0	0	0	0	0	0	0	0	0	1 (100.0%)	1 (2.2%)
Confusional state	0	0	0	0	0	0	0	0	0	0	1 (25.0%)	0	1 (2.2%)
Rash	1 (25.0%)	0	0	0	0	0	0	0	0	0	0	0	1 (2.2%)
Sepsis	0	0	0	0	0	1 (16.7%)	0	0	0	0	0	0	1 (2.2%)
**Adverse events leading to dose reduction of APVO436**													
Chills	0	0	0	0	0	1 (16.7%)	0	0	0	0	0	0	1 (2.2%)
Cytokine release syndrome	0	0	0	0	0	1 (16.7%)	0	0	0	0	0	0	1 (2.2%)
**Adverse events leading to permanent discontinuation of APVO436**													
Confusional state	0	0	0	1 (16.7%)	0	0	0	0	0	0	0	1 (100.0%)	2 (4.3%)
Amnesia	0	0	0	0	0	0	0	0	0	0	0	1 (100.0%)	1 (2.2%)
Asthenia	0	0	0	0	0	1 (16.7%)	0	0	0	0	0	0	1 (2.2%)
Cytokine release syndrome	0	0	0	1 (16.7%)	0	0	0	0	0	0	0	0	1 (2.2%)
Delirium	0	0	0	0	0	1 (16.7%)	0	0	0	0	0	0	1 (2.2%)
Neurotoxicity	0	0	0	0	0	0	0	0	0	0	0	1 (100.0%)	1 (2.2%)
Pneumonia	0	0	0	0	0	1 (16.7%)	0	0	0	0	0	0	1 (2.2%)
Pyrexia	0	0	0	1 (16.7%)	0	0	0	0	0	0	0	0	1 (2.2%)
Sepsis	0	0	1 (33.3%)	0	0	0	0	0	0	0	0	0	1 (2.2%)
Shock	0	0	0	1 (16.7%)	0	0	0	0	0	0	0	0	1 (2.2%)
Supraventricular tachycardia	0	0	0	0	0	1 (16.7%)	0	0	0	0	0	0	1 (2.2%)

MedDRA PT	Cohorts												
	**DL-1 Dose: 0.3 mcg/week (*N* = 4)**	**DL-2 Dose: 1 mcg/week (*N* = 3)**	**DL-3 Dose: 3 mcg/week (*N* = 3)**	**DL-4 Dose: 9 mcg/week (*N* = 6)**	**DL-5 Dose: 12 mcg/week (*N* = 3)[a]**	**DL-6A Dose: 18 mcg/week (*N* = 6)[b]**	**DL-6B Dose: 12 mcg/week (*N* = 3)[c]**	**DL-7 Dose: 24 mcg/week (*N* = 4)[d]**	**DL-8[2] Dose: 36 mcg/week (*N* = 6)[e]**	**DL-9 Dose: 48 mcg/week (*N* = 3)[f]**	**DL-10 Dose: 60 mcg/week (*N* = 4)[g]**	**DL-A[2] Dose: 36 mcg/week (*N* = 1)[h]**	**Total *N* = 46 *n* (%)**
Tumor lysis syndrome	0	0	0	1 (16.7%)	0	0	0	0	0	0	0	0	1 (2.2%)

CTCAE: Common terminology criteria for adverse events; MedDRA: Medical dictionary for regulatory activities; PT: Preferred term; N: Total number of patients; n: number of patients with event

[a] Cohort 5: 12 mcg/week [weekly step dosing in Cycle 1: 6, 9, 12, 12 mcg]

[b] Cohort 6A: 18 mcg/week [weekly step dosing in Cycle 1: 6,9,12,18 mcg]

[c] Cohort 6B: 12 mcg/week [weekly step dosing in Cycle 1: 6,12,12,12 mcg]

[d] Cohort 7: 24 mcg/week [weekly step dosing in Cycle 1: 6, 12, 18, 24 mcg]

[e] Cohort 8: 36 mcg/week [weekly step dosing in Cycle 1: 6,12,18, 36 mcg]

[f] Cohort 9: 48 mcg/week [weekly step dosing in Cycle 1: 6,12,18,48 mcg]

[g] Cohort 10: 60 mcg/week [weekly step dosing in Cycle 1: 6,12,18,60 mcg]

[h] Cohort A: 36 mcg/week [Daily step dosing in week 1: 6, 9, 12,18 mcg; in week 2:18 mcg × 2; in week 3 and 4:36 mcg × 2]

Note:

1. When the same event was reported twice for the same patient, it was only counted once, and the highest grade (worst grade) was captured.

Patient 219-0005 was originally assigned to treatment with APVO436 according to Cohort A schedule, received 6 mcg of APVO436 on day 1 and developed Grade 3 CRS during the second infusion. The cumulative dose infused was 8.5 mcg. CRS was complicated by non-ST elevation myocardial infarction (N-STEMI). Patient's CRS and cardiac function fully recovered, and patient was switched to the weekly regimen and entered into Cohort 8. All AEs for 219-0005 are captured under Cohort 8.

Table S5. Listing of Dose Limiting Toxicities (DLTs) in Study 5001.

Patient No.	Cohort#	Preferred term (CTCAE Grade)	System Organ Class	Outcome	Relatedness with APVO436	Action Taken with APVO436	Is the Adverse Event serious?
217-0002	DL-4[a]	Acute respiratory failure (Grade 4)	Respiratory, thoracic and mediastinal disorders	Recovered or Resolved	Related	Dose Not Changed	Yes
		Shock (Grade 4)	Vascular disorders	Recovered or Resolved	Related	Drug Permanently Discontinued	No
219-0005	DL-A[b] /DL-8[c]	Cytokine release syndrome (Grade 3)	Immune system disorders	Recovered or Resolved	Related	Drug Interrupted	Yes

AE: Adverse event; CRS: Cytokine release syndrome; CTCAE: Common terminology criteria for adverse events; DL: Dose level; DLT: Dose limiting Toxicity; N-STEMI: Non-ST elevation myocardial infarction

[a]: Dose: 9 mcg/week

[b]: Cohort 8: 36 mcg/week [weekly step dosing in Cycle 1: 6,12,18, 36 mcg]

[c]: Cohort A: 36 mcg/week [Daily step dosing in week 1: 6, 9, 12,18 mcg; in week 2:18 mcg × 2; in week 3 and 4:36 mcg × 2]

Patient 219-0005 was originally assigned to treatment with APVO436 according to Cohort A schedule, received 6 mcg of APVO436 on day 1 and developed Grade 3 CRS during the second infusion. The cumulative dose infused was 8.5 mcg. CRS was complicated by non-ST elevation myocardial infarction (N-STEMI). Patient's CRS and cardiac function fully recovered, and patient was switched to the weekly regimen and entered into Cohort 8. All AEs for 219-0005 are captured under Cohort 8 in all tables in this paper except in this table as the DLT occurred while he was in Cohort A.

Table S6. CRS-Associated Changes in Serum Cytokine Levels of APVO436 Treated R/R AML/MDS Patients.

Cytokine	Pre-Treatment	Day 1–2	Day 3–6	Day 7–11
IL-6, pg/mL	Mean: 5.2 ± 0.6<br>Median: 6.0<br>Range: 3.7–6.4	Mean: 755.0 ± 393.6<br>Median: 558.0<br>Range: 1.2–2103.0	Mean: 430.7 ± 143.2<br>Median: 531.4<br>Range: 12.0–648.0	Mean: 108.2 ± 43.3<br>Median: 80.2<br>Range: 39.0–233.4
IFN-g pg/mL	Mean: 12.9 ± 5.2<br>Median: 15.4<br>Range: 2.9–20.3	Mean: 3.3 ± 1.6<br>Median: 3.3<br>Range: 1.7–4.8	Mean: 15.7 ± 11.0<br>Median: 15.7<br>Range: 4.7–26.6	Mean: 4.9 ± 1.2<br>Median: 6.0<br>Range: 2.5–6.3
IL-10, pg/mL	Mean: 1.4 ± 0.4<br>Median: 0.9<br>Range: 0.7–2.7	Mean: 1.3 ± 0.3<br>Median: 1.1<br>Range: 0.8–2.1	Mean: 4.4 ± 2.8<br>Median: 1.8<br>Range: 1.3–10.0	Mean: 1.0 ± 0.2<br>Median: 1.0<br>Range: 0.7–1.3
IL-17A, pg/mL	Mean: 4.1 ± 1.8<br>Median: 2.9<br>Range: 0.8–11.2	Mean: 3.3 ± 1.8<br>Median: 1.8<br>Range: 1.1–8.7	Mean: 0.4 ± 0.0<br>Median: 0.4<br>Range: 0.4–0.4	Mean: n/a<br>Median: n/a<br>Range: n/a
IL-5, pg/mL	Mean: 1.5 ± 1.0<br>Median: 0.5<br>Range: 0.5–5.4	Mean: 5.8 ± 4.4<br>Median: 1.9<br>Range: 0.3–23.2	Mean: 0.9 ± 0.3<br>Median: 1.0<br>Range: 0.2–1.5	Mean: n/a<br>Median: n/a<br>Range: n/a
MCP-1, pg/mL	Mean: 168.2 ± 23.4<br>Median: 162.2<br>Range: 108.3–230.2	Mean: 276.8 ± 54.3<br>Median: 254.1<br>Range: 121.6–426.1	Mean: 438.8 ± 209.2<br>Median: 438.8<br>Range: 229.6–647.9	Mean: 130.6 ± 26.6<br>Median: 105.6<br>Range: 102.4–183.8
TNFa, pg/mL	Mean: 3.8 ± 1.1<br>Median: 4.9<br>Range: 0.7–6.4	Mean: 4.9 ± 2.7<br>Median: 3.1<br>Range: 0.9–12.4	Mean: 2.4 ± 0.5<br>Median: 2.8<br>Range: 1.4–2.9	Mean: 1.5 ± 0.3<br>Median: 1.6<br>Range: 1.0–1.9
